# Clinical efficacy of linagliptin combined with irbesartan in patients with diabetic nephropathy

**DOI:** 10.12669/pjms.38.1.4417

**Published:** 2022

**Authors:** Jie Liu, Jing Zhang, Ming-hui Hou, Wei-xuan Du

**Affiliations:** 1 Jie Liu, Department of Endocrinology, Affiliated Hospital of Hebei University, Baoding 071000, Hebei, China; 2 Jing Zhang, Department of Cardiology, Affiliated Hospital of Hebei University, Baoding 071000, Hebei, China; 3 Ming-hui Hou, Department of Endocrinology, Affiliated Hospital of Hebei University, Baoding 071000, Hebei, China; 4 Wei-xuan Du Department of Education, Affiliated Hospital of Hebei University, Baoding 071000, Hebei, China

**Keywords:** Diabetic nephropathy, Linagliptin, Irbesartan

## Abstract

**Objective:**

To determine the clinical efficacy of linagliptin combined with irbesartan in patients with diabetic nephropathy (DN).

**Methods:**

Seventy-two patients who were admitted to our department of endocrinology in our hospital during January 2018 and June 2019 were randomly divided into a control group (administered with irbesartan only, n=36) and a treatment group (treated with irbesartan and linagliptin, n=36). The course of treatment lasted for three months. FBG (fasting blood glucose), 2hPBG (2h postprandial blood sugar), HbA1C (hemoglobin A1c), Cys-C (cystatin C), SCr (serum creatinine), BUN (blood urea nitrogen), UACR (urine albumin-to-creatinine ratio), CRP (C-reactive protein), IL-6 (interleukin-6), and SOD (superoxide dismutase) were tested pre- and post-treatment to evaluate the clinical efficacy and adverse effects of the two treatment plans after three months of treatment.

**Results:**

Compared with the pre-treatment levels, FBG, 2hPBG, HbA1c, Cys-C, SCr, BUN, UACR, CRP, IL-6, and SOD in both groups were significantly improved following the three-month treatment (P<0.05, respectively). Post-treatment levels of FBG, 2hPBG, HbA1c, Cys-C, SCr, BUN, UACR, CRP, and IL-6 in the treatment group were significantly lower than in the control group (P<0.05, respectively), while the treatment group exhibited a higher level of SOD compared with the control group (P<0.05). No serious adverse reaction occurred in either group (P>0.05).

**Conclusion:**

Combined-modality treatment with linagliptin and irbesartan shows favorable clinical efficacy in treating diabetic nephropathy as it effectively protects the kidneys and improves kidney function by inhibiting inflammatory and oxidative stress responses.

## INTRODUCTION

At present, there are more than 400 million individuals living with diabetes, and the number keeps increasing, making diabetic nephropathy (DN), one of the common complications of diabetes, a global public health challenge.^1^ With the number of diabetic patients growing, the incidence of DN rises dramatically.^2^ DN is one of the most common and serious chronic microvascular complications occurring in those with diabetes, as well as a major cause of chronic and end-stage kidney diseases^3^,^4^, which brings a heavy financial burden to both the society and the patients’ families. Against this backdrop, it has become a popular interest of research to develop new medications that improve the long-term prognosis of DN. Linagliptin is one of the next-generation dipeptidyl peptidase (DPP)-4 inhibitors. Studies have shown that DPP-4 inhibitors enable kidney protection as they mediate a reduction in urine protein by reducing inflammatory and oxidative stress responses.^5^ Irbesartan is an angiotensin II receptor blocker (ARB) that has been proved to exhibit beneficial effects on blood pressure (BP) and inflammation.^6^ However, the protective effect of the combination of the linagliptin and irbesartan on the kidney has rarely been studied. In this study, DN was treated with linagliptin in combination with irbesartan, aiming to observe the renal protective effects of these drugs in addition to blood glucose (BG) and BP control. The purpose of this study was to explore the clinical value of linagliptin combined with irbesartan in the treatment of diabetic nephropathy. And to add a persuasive clinical data for the treatment of diabetic nephropathy.

## METHODS

This study involved 72 DN patients who were admitted to our hospital from January 2018 to June 2019, and the patients were selected according to the principle of random sampling, and were divided into a control group (n=36) and a treatment group (n=36) using a random number table. With the approval granted by the hospital’s ethical committee, this study was completed successfully.

### Ethical approval:

The study was approved by the Institutional Ethics Committee of Affiliated Hospital of Hebei University, and written informed consent was obtained from all participants

### Inclusion criteria:

A patient was rendered eligible for the clinical trial if he/she

• was 18 to 70 years old;
• never had used any DDP-4 or angiotensin-converting enzyme (ACE) inhibitors, met the DN diagnostic criteria^7^: Patients with diabetes have decreased glomerular filtration rate or have been found to have albuminuria, and confirmed by renal needle biopsy;
• Agreed to participate in this study voluntarily with informed consent.

### Exclusion criteria:

A patient was ineligible to participate in this study if he/she met any of the following criteria:

• kidney disease not caused b
• Patients with contraindications to the use of relevant drugs;
• pregnancy or breast-feeding;
• mental illness.

The control group consisted of 19 male and 17 female patients, with the mean age of (41.28±11.16) years (range: 35-67), mean course of disease of (10.27±6.81) years, and mean BMI of (23.56±2.17) kg/m^2^. The treatment group included 20 males and 16 females, with the mean age of (41.89±12.26) years (range: 33-69), mean course of disease of (11.04±6.92) years, and mean BMI of (24.32±2.21) kg/m^2^. There was no statistically significant difference in demographic characteristics between the two groups (P>0.05, respectively).

### Treatment methods:

Based on routine management of diabetes (including a diabetes-specific diet, exercise education, BP and BG control, and treatment of lipid metabolism disorders), the control group was treated with 150 mg/d oral irbesartan, and the treatment group was commenced on the combined-modality treatment (CMT) with 150 mg/d oral irbesartan and 5 mg/d oral linagliptin. Both groups were under close observation for 90 days.

Outcome measures Parameters related to blood glucose, kidney function, inflammation, and oxidative stress were used for the evaluation of clinical outcomes. FBG (fasting blood glucose), 2hPBG (2h postprandial blood sugar), HbA1C (hemoglobin A1c), SCr (serum creatinine), BUN (blood urea nitrogen), Cys-C (cystatin C), UACR (urine albumin-to-creatinine ratio), CRP (C-reactive protein, measured by ELISA (enzyme-linked immunosorbent assay)), IL-6 (interleukin-6), and SOD (superoxide dismutase, determined by a pyrogallol system) were tested by our laboratory department.

### Data processing:

SPSS 20.0 was used for data processing. Measurement data were expressed by (X̄±s), with intragroup comparison before and after the three-month treatment being examined by the t-test, and intergroup comparison by the dependent t-test for paired samples. Enumeration data were represented by (%) and examined by the chi-squared test. A 95% confidence interval was used. P<0.05 was considered indicative of a significant difference.

## RESULTS

After the treatment, FBG, 2hPBG, and HbA1c in both groups were improved compared to the pre-treatment levels, and the differences were statistically significant (P<0.05); the FBG, 2hPBG, and HbA1c levels in the treatment group were lower than in the control group, which indicated differences of statistical significance (P<0.05). Table-I.

**Table I:** Comparison of pre- and post-treatment FBG, 2hPBG, and HbA1c levels in and between the treatment and control groups (x̅ ±s, n=36).

Group	Period	FBG (mmol/L)	2hPBG (mmol/L)	HbA1C (%)
Control group	Pre-treatment	10.55±1.49	13.55±1.63	7.91±0.86
	Post-treatment	8.53±1.24^Δ^	10.28±1.41^Δ^	7.06±0.66^Δ^
Treatment group	Pre-treatment	10.20±1.32	13.89±1.70	7.88±0.82
	Post-treatment	7.62±1.10^*Δ^	9.18±1.32^*Δ^	6.65±0.53^*Δ^

ΔP<0.05 compared with the pre-treatment levels;

* P<0.05 compared with the control group.

Compared with the pre-treatment levels of SCr, BUN, Cys-C, and UACR, both groups showed a marked improvement in kidney function, and the differences had statistical significance (P<0.05); following the three-month treatment, the SCr, BUN, Cys-C, and UACR levels in the treatment group became lower than in the control group, and the differences were statistically significant (P<0.05). Table-II.

**Table II:** Comparison of pre- and post-treatment SCr, BUN, Cys-C, and UACR levels in and between the treatment and control groups (x̅ ±s, n=36).

Group	Period	Cys-C(mg/L)	UACR (mg/g)	SCr (umol/L)	BUN (mmol/L)
Control group	Pre-treatment	1.74±0.68	120.73±10.73	110.24±13.21	8.21±1.18
	Post-treatment	1.40±0.50^Δ^	81.42±9.46^Δ^	87.11±12.31^Δ^	6.65±1.14^Δ^
Treatment group	Pre-treatment	1.65±0.62	129.26±11.55	113.43±14.51	8.33±1.09
	Post-treatment	1.21±0.39^*Δ^	62.23±8.25^*Δ^	74.65±10.17^*Δ^	5.80±1.01^*Δ^

ΔP<0.05 compared with the pre-treatment levels;

* P<0.05 compared with the control group.

Comparing the pre- and post-treatment CRP, IL-6, and SOD levels, both groups exhibited a substantial improvement, with statistically significant differences (P<0.05); the post-treatment CRP and IL-6 levels in the treatment group were significantly lower than in the control group, whereas the treatment group had a higher level of SOD compared with the control group, and the differences were statistically significant (P<0.05, respectively). Table-III.

**Table III:** Comparison of pre- and post-treatment CRP, IL-6, and SOD in and between the treatment and control groups (x̅ ±s, n=36).

Group	Period	SOD (ug/mL)	CRP (mg/L)	IL-6 (mg/L)
Control group	Pre-treatment	53.62±13.41	14.55±3.93	6.81±2.06
	Post-treatment	89.53±19.44^Δ^	7.98±2.14^Δ^	5.96±1.61^Δ^
Treatment group	Pre-treatment	51.12±12.54	13.89±4.15	6.79±2.01
	Post-treatment	98.62±20.64^*Δ^	5.58±1.90^*Δ^	4.72±1.52^*Δ^

ΔP<0.05 compared with the pre-treatment levels;

* P<0.05 compared with the control group.

### Adverse reactions:

Comparing the adverse reactions occurring in the patients of both groups, no statistically significant difference was detected between these two groups (P<0.05). Table-IV.

**Table IV:** Comparison of adverse reactions between the treatment and control groups (x̅ ±s, n=36).

Group	Nausea	Headache	Diarrhea	Total
Control group	1 (2.8)	1 (2.8)	0 (0)	4 (5.6)
Treatment group	2 (5.6)	0 (0)	1 (2.8)	5 (8.3)

## DISCUSSION

With the ongoing changes in lifestyles and population aging in China, the incidence of DN has increased dramatically.^8^ With the disease having a complex pathogenesis, it is widely accepted that the development and progression of DN have very close correlations with inflammatory response, oxidative stress, lesions in the immune and endocrine systems, endothelial dysfunction, abnormal lipid metabolism, and hypertension.^9^,^10^ DN is an important risk factor for end-stage kidney disease and death in patients with diabetes.^11^ The medical community and other researchers from the academic community have directed close attention to the development of scientific and effective treatment for DN patients.

Linagliptin is a novel DDP-4 inhibitor that helps lower the blood sugar level. As the only medication that depends on enterohepatic circulation for elimination, linagliptin can be used in patients with chronic kidney disease without dose adjustments, which indicates good safety for DN treatment.^12^ Linagliptin is effective in reducing glomerulosclerosis, renal oxidative stress, and UACR probably by inhibiting the RAAS (renin-angiotensin-aldosterone system) and DPP-4 activity.^13^,^14^ Observation studies have found that in CD-1 mouse models with DN, linagliptin plays a role in resisting oxidation, inflammation, and fibrosis, destroying the advanced glycation endoproduct (AGE)–RAGE signaling pathway and raising the levels of GLP-1 and SDF-1, thereby improving endothelial dysfunction and providing multi-level kidney protection.^15^,^16^ Irbesartan is a common depressor for clinical use as it is safe and effective in lowering high blood pressure. Additionally, irbesartan, as an ARB, also helps relieve proteinuria and delay glomerulosclerosis and tubulointerstitial fibrosis by regulating protein kinases C (PKC) and RAS (renin-angiotensin system) activity, inhibiting inflammatory response and reducing oxidative stress and injury to Sertoli cells.^17^,^18^

Persistent chronic inflammatory response is a critical mechanism that denotes DN development and progression. CRP is a nonspecific acute phase reactant that exhibits a significant increase in response to acute/chronic inflammation and stress.^19^ Increased CRP triggers oxidative-stress response and induces direct glomerular endothelial injury, leading to capillary dilation in renal microcirculation and an increase in urine albumin. IL-6 is an anti-inflammatory mediator. Increased IL-6 can induce microangiopathic changes in glomeruli, add lipid peroxidation metabolites to the body and accelerate DN development and progression.^20^ Studies have demonstrated that oxidative stress response plays a vital role in the pathogenesis of DN as oxidative stress is involved in almost all pathological changes in DN, including signal transduction of RAS, chronic inflammation, basement membrane thickening in glomeruli and renal tubules, Sertoli cell dysfunction, and renal fibrosis. SODs are a group of antioxidant enzymes that provide antioxidant defense by eliminating free radicals in the body and maintain the oxidant/antioxidant balance and normal vascular endothelial function. With SOD being a decisive player in structural and functional protection for the kidneys, decreased SOD is considered to be associated with renal function impairment.^21^ Cys-C, a secretory basic protein, is a well-recognized sensitive serum indicator of kidney function in the clinical setting. In human bodies, Cys-C can only be metabolized by the kidneys as it passes through the glomerular filtration membrane and is reabsorbed and metabolized by renal tubules. In the case of impaired renal function, an increase in the Cys-C level can be observed due to abnormal Cys-C degradation.^22^,^23^

This study showed that after the three-month treatment, FBG, 2hPBG, HbA1C, SCr, BUN, Cys-C, and UACR in both groups decreased remarkably compared with the pre-treatment levels, with the treatment group showing a more significant reduction in these parameters. This reveals that both linagliptin and irbesartan can reduce urine protein and improve kidney function, and the combined modality treatment with these drugs can deliver more effective outcomes. Comparing the pre- and post-treatment levels of CRP, IL-6, and SOD, significant improvement was seen in both groups after treatment. The post-treatment CRP and IL-6 levels in the treatment group were significantly lower than in the control group, whereas the post-treatment SOD level in the treatment group was significantly higher than in the control group (P<0.05, respectively). This indicates that linagliptin and irbesartan probably provide kidney protection by inhibiting inflammatory and oxidative stress responses.

### Limitations of the study:

The number of subjects included in this study is limited. In addition, we only analyzed the cases included in our hospital, which may not be representative enough. We look forward to a multi-center study in the future to reach more comprehensive conclusions.

## CONCLUSION

Since inflammatory and oxidative stress responses play an important role in DN development and progression, the combined modalities of linagliptin and irbesartan, independent of their hypoglycemic and hypotensive effects, appear to protect kidney function by reducing inflammatory factors and products of oxidative stress and thereby produce desirable outcomes in the clinical treatment of DN.

### Authors’ Contributions:

**JL & JZ:** Designed this study, prepared this manuscript, are responsible and accountable for the accuracy and integrity of the work.

**MHH:** Collected and analyzed clinical data.

**WXD:** Significantly revised this manuscript.
